# Prevalence and risk factors predisposing low bone mineral density in patients with thalassemia

**DOI:** 10.3389/fendo.2024.1393865

**Published:** 2024-06-24

**Authors:** Natnicha Ananvutisombat, Adisak Tantiworawit, Teerachat Punnachet, Nonthakorn Hantrakun, Pokpong Piriyakhuntorn, Thanawat Rattanathammethee, Sasinee Hantrakool, Chatree Chai-Adisaksopha, Ekarat Rattarittamrong, Lalita Norasetthada, Kanda Fanhchaksai, Pimlak Charoenkwan

**Affiliations:** ^1^ Department of Internal Medicine, Faculty of Medicine, Chiang Mai University, Chiang Mai, Thailand; ^2^ Division of Hematology, Department of Internal Medicine, Faculty of Medicine, Chiang Mai University, Chiang Mai, Thailand; ^3^ Thalassemia and Hematology Center, Faculty of Medicine, Chiang Mai University, Chiang Mai, Thailand; ^4^ Division of Hematology and Oncology, Department of Pediatrics, Faculty of Medicine, Chiang Mai University, Chiang Mai, Thailand

**Keywords:** thalassemia, Low BMD, z score, transfusion dependency, IGF-1, hypogonadism

## Abstract

**Background:**

A common complication of thalassemia is secondary osteoporosis. This study aimed to assess the prevalence and factors associated with low BMD in thalassemic patients.

**Method:**

This is a cross-sectional study. Eligible patients were males aged within 18–49 years or premenopausal women diagnosed with thalassemia in Chiang Mai University Hospital between July 2021 and July 2022. The diagnosis of low BMD by dual-energy x-ray absorptiometry (DXA) was defined as a Z-score of -2.0 SD or lower in either the lumbar spine or femoral neck. Clinical factors associated with low BMD were analyzed using a logistic regression model.

**Results:**

Prevalence of low BMD was 62.4% from 210 patients with a mean age of 29.7 ± 7.6 years. The predominant clinical characteristics of low BMD thalassemia patients were being female, transfusion-dependent (TDT) and a history of splenectomy. From multivariable analysis, the independent variables associated with low BMD were transfusion dependency (odds ratio, OR 2.36; 95%CI 1.28 to 4.38; p=0.006) and body mass index (BMI) (OR 0.71; 95%CI 0.61 to 0.82; p<0.001). Among patients with low BMD, we observed a correlation between a Z-score with low IGF-1 levels (β=-0.42; 95% CI -0.83 to -0.01; p=0.040), serum phosphate levels (β=0.40; 95% CI 0.07 to 0.73; p=0.016) and hypogonadism (β=-0.48, 95% CI -0.91 to -0.04, p=0.031).

**Conclusion:**

This study found a prevalence of low BMD in 62.4% of subjects. Factors associated with low BMD were TDT and BMI. Within the low BMD subgroup, hypogonadism, serum phosphate and low serum IGF-1 levels were associated with a lower Z-score.

## Introduction

Thalassemia results from a defect in hemoglobin synthesis, leading to chronic anemia and ineffective red blood cell formation (1). Thalassemia has currently been categorized into transfusion-dependent thalassemia (TDT) and non-transfusion-dependent thalassemia (NTDT). This distinction is considered beneficial for the purposes of treatment planning (2).

One of the common complications associated with thalassemia is secondary osteoporosis. According to a Taiwanese database, 17% of thalassemia major patients had osteoporosis (3). In northeastern Thailand, the prevalence of fractures, which are a complication of osteoporosis, was 13.0–16.3% among thalassemic patients. The characteristics associated with vertebral fracture were age, low bone mineral density (BMD), low trabecular bone score (TBS), history of splenectomy and endocrinopathy (4, 5). The most common fracture sites were L1 and T12 (4).

Low BMD in thalassemic patients was found to occur at ages earlier than 65 years old, which is the screening age for the general population (4, 5). Nowadays, the non-invasive tool used to diagnose osteoporosis via BMD is dual-energy x-ray absorptiometry (DXA) (6). According to the World Health Organization (WHO), osteoporosis in postmenopausal women is defined as a T-score of -2.5 SD or lower in those aged more than 50years old. However, the T-score is not aplicable to men younger than 50 years old or premenopausal women. In our study, Z-score was indicative of a BMD value that could be compared to an aged matched population. Accordingly, this was more applicable for use in this study population, especially in cases of thalassemia patients who were determined to be younger than the expected age of onset. A Z-score of -2.0 SD or lower is defined as a BMD score below the expected range for the subject’s age (7). In a previous study, Tzoulis et al. defined osteoporosis as a Z-score <-2.0 in patients aged over 18 years old with beta-thalassemia (8). Additionally, Vogiatzi MG, et al. defined osteoporosis as a Z-score <-2.0 and osteopenia as a Z-score between -1.0 and -2.0 in patients aged from 6 to 75 years. This included both beta and alpha-thalassemia patients (9). However, various studies have defined osteoporosis as a Z-score <-2.5 and osteopenia as a Z-score between -1.0 to -2.5 (10–13). Thus, there has been no established definition of osteoporosis in particular groups, especially among the thalassemic population.

To date, osteoporosis is perceived as a multifactorial issue. Each study reports different factors. Therefore, recognition of the significant risk factors of osteoporosis is crucial for identification of high-risk patients. Features previously discovered to have a role in bone metabolism include serum ferritin, type of iron chelator, zinc levels, levels of insulin-like growth factor-1 (IGF-1), hypothyroidism and calcium levels, physical activity and high caloric intake measured indirectly by body mass index (BMI) (14–17). However, the relationships between IGF-1, hematocrit, vitamin D and BMD are still being debated (15–18).

The proportion of alpha-thalassemia cases in our region was higher when compared with other regions included in previous studies. The majority of the previous studies were predominantly focused on beta-thalassemia. Therefore, the objective of this study was to determine the prevalence of low BMD and to identify the risk factors of low BMD in thalassemic patients, which could then be applied to predict the probability of osteoporosis. This would help in the selection of at-risk populations for early detection of osteoporosis, especially in resource-limited countries.

## Methods

### Study design

This was a single-center, cross-sectional, retrospective study that used data obtained from the thalassemia clinic located in Chiang Mai University Hospital. The data were obtained from electronic medical records. The study was approved by the Institutional Research Ethics Committee at the Faculty of Medicine, Chiang Mai University (Study number: MED-2564–08128).

### Definition

Low BMD was defined as a Z-score of -2.0 or lower at either the lumbar spine or femoral neck, as measured by DXA. Normal BMD is defined as a Z-score higher than -2.0 at both the lumbar spine and femoral neck.

Hypogonadism was characterized by low levels of sex steroids according to age-specific reference ranges, with or without symptoms such as delayed puberty (no testicular enlargement by the age of 14 in boys and the absence of breast development by the age of 13 in girls), infertility, and decreased libido in men, and irregular or absent menstrual periods, hot flashes, and infertility in women. Both hypergonadotropic (low sex hormones, high gonadotropins), and hypogonadotropic (low sex hormones and gonadotropins) hypogonadism fell under this condition.

Adrenal insufficiency was defined by low morning cortisol levels and confirmed by peak cortisol below 18 mcg/dL after adrenocorticotropic hormone (ACTH) stimulation.

Hypothyroidism was defined by high thyroid stimulating hormone (TSH) or low free thyroxine (FT4) compared to age-specific reference ranges.

Hypoparathyroidism was defined by low serum calcium and high serum phosphate and confirmed by low parathyroid hormone.

### Study population

Eligible patients were aged ≥ 18 years, diagnosed with thalassemia using the high-performance liquid chromatography (HPLC) method and those who had received treatment at the thalassemia clinic of Chiang Mai University Hospital during the period between July 2021 and July 2022. In cases of multiple visits, the visit associated with the least amount of missing data was chosen.

Patients were excluded if DXA was not done, if they were aged 50 years or older, were menopausal women, pregnant or had undergone prior stem cell transplantation, secondary osteoporosis, and osteomalacia. Osteomalacia was excluded by clinical and vitamin D level.

The clinical data exported from the electronic medical records included demographic data, comorbidities, type of thalassemia and DXA data. DXA data measured by dual-energy X-ray absorptiometry (DXA; Hologic Discovery A, Hologic Company, Marlborough, MA, USA). Laboratory data were as follows: CBC, FBS, HbA1C, albumin, ferritin, calcium, phosphate, vitamin D levels, PTH, FT4, FT3, TSH, LH, estradiol, testosterone, prolactin, serum IGF-1 levels, HBsAg, anti-HCV and anti-HIV.

### Outcomes

The primary outcome was to determine the prevalence of low BMD in thalassemic patients. The secondary outcome was to identify risk factors associated with low BMD scores in this population.

### Statistical analysis

As has been reported in previous studies, the prevalence of low BMD in thalassemic participants was approximately 50–60% (8, 9, 19, 20). Assuming a prevalence rate of 50% with an effect size of 10%, 95% level of significance and 80% power of test, the required total sample size would be at least 194.

Categorical data were reported as numbers and percentages. Continuous data were reported as mean and standard deviations, or median with interquartile range depending on data distribution.

Each covariate and outcome was examined using a univariate logistic regression model. The selected covariates with established clinical significance, which were statistically significant with a p-value less than 0.1, were analyzed with a multivariable logistic regression model. The association was determined using an odds ratio (OR).

Additional analyses were conducted within the low BMD subgroup using a linear regression model to demonstrate the degree of correlation between each hormonal laboratory and a Z-score. All analyses were conducted using Stata version 17 (StataCorp LLC, USA).

## Results

### Study population

A total of 210 patients were enrolled in this study, while 131 were identified with low BMD. The clinical characteristics of low BMD thalassemia patients were predominantly female (56.5%), transfusion-dependent (68.7%), those who had undergone previous splenectomy (66.4%) and those who had a mean age of 29.7 ± 7.6 years. The majority of subjects (86.2%) had at least one abnormal hormonal function. The results showed 32 patients (15.2%) had hypogonadism, 22 of 32 patients (68.8%) were taking hormone replacement therapy, 23 patients (10.9%) had hypothyroidism, 16 patients (7.6%) had adrenal insufficiency, the dose of steroid treatment was mainly prednisolone with the dose of 2.5 to 5 mg per day, and 10 patients (4.8%) had hypoparathyroidism. The prevalence of low BMD was 62.4%. Other comorbidities and laboratory results are shown in **Table 1**.

**Table 1:** Baseline characteristics.

	Total, n (%) (N= 210)	Low BMD (n= 131)	Normal BMD (n= 79)
Demographic data			
**Age (years), mean ± sd**	31.5 ± 8.5	29.7 ± 7.6	34.4 ± 9.3
Gender n(%)			
• **Male**	83 (39.5)	57 (43.5)	26 (32.9)
• **Female**	127 (60.5)	74 (56.5)	53 (67.1)
**BMI (kg/m^2^), mean ± sd, n= 207**	19.4 ± 2.3	18.8 ± 2.1	20.4 ± 2.2
Type of thalassemia n(%)			
• **TDT**	128 (60.9)	90 (68.7)	38 (48.1)
• **NTDT**	82 (39.1)	41 (31.3)	41 (51.9)
• **Alpha thalassemia**	38 (18.1)	18 (13.7)	20 (25.3)
• **Beta thalassemia**	172 (81.9)	113 (86.3)	59 (74.7)
Splenectomy n(%)			
• **Yes**	115 (54.8)	87 (66.4)	28 (35.4)
• **No**	95 (45.2)	44 (33.6)	51 (64.6)
Smoking, n= 156 n(%)			
• **Yes**	3/156 (1.9)	3 (2.9)	0 (0)
• **No**	153/156 (98.1)	99 (97.1)	54 (100)
Alcohol drinker, n= 157 n(%)			
• **Yes**	5/157 (3.2)	4 (3.9)	1 (1.8)
• **No**	152/157 (96.8)	98 (96.1)	54 (98.2)
Comorbidity n(%)			
• **Abnormal hormone** ** (Hypogonadism, Hypothyroid,** ** Adrenal insufficiency)**	157/176 (89.2)	100/116 (86.2)	57/60 (95.0)
• **Non-communicating disease** ** (HTN, DM)**	15/210 (7.1)	11/131 (8.4)	4/79 (5.1)
• **Chronic viral hepatitis** ** infection (HBV, HCV)**	22/192 (11.5)	15/124 (12.1)	7/68 (10.3)
• **Pulmonary hypertension**	18/210 (8.6)	14/131 (10.7)	4/79 (5.1)
Iron chelation n(%)			
• **Yes**	178 (84.8)	116 (88.6)	62 (78.5)
• **No**	32 (15.2)	15 (11.4)	17 (21.5)
Steroid			
• **Yes**	22 (10.5)	18 (13.7)	4 (5.1)
• **No**	188 (89.5)	113 (82.3)	75 (94.9)
Blood examination			
• **Hemoglobin (g/dl),** ** mean ± sd**	7.46 ± 1.21	7.33 ± 1.29	7.69 ± 1.04
• **Hematocrit (%), mean ± sd**	24.57 ± 4.37	24.12 ± 4.59	25.32 ± 3.90
• **Creatinine (mg/dl),** median ** [IQR], n= 206**	0.52 [0.43, 0.63]	0.51 [0.41, 0.60]	0.54 [0.45, 0.70]
• **Ferritin (mg/L),** ** median[IQR]**	1.07 [0.67, 2.35]	1.14 [0.76, 2.38]	1.00 [0.62, 2.35]
• **Serum albumin (g/dl),** ** mean ± sd, n= 209**	4.38 ± 0.46	4.33 ± 0.49	4.48 ± 0.39
• **Calcium (mg/dl), mean ± sd,** ** n= 167**	9.01 ± 0.48	8.99 ± 0.49	9.07 ± 0.43
• **Phosphate (mg/dl),** ** mean ± sd, n= 165**	4.00 ± 0.75	4.08 ± 0.71	3.77 ± 0.84
• **Vitamin D level (ng/ml),** ** mean ± sd, n= 107**	28.67 ± 10.89	28.61 ± 9.64	28.85 ± 13.90
• **PTH (pg/ml), median[IQR],** ** n= 79**	25.32 [18.30, 35.94]	25.30 [18.30, 34.99]	33.51 [24.45, 46.53]
• **IGF-1 (ng/ml), median[IQR],** ** n= 80**	65.65 [39.15, 95.40]	65.60 [38.75, 95.40]	76.85 [58.85, 105.80]
History of extramedullary hematopoiesis, n(%)			
• **Yes**	20 (9.5)	14 (10.7)	6 (7.6)
• **No**	190 (90.5)	117 (89.3)	73 (92.4)

BMI, Body mass index; TDT, Transfusion dependent thalassemia; NTDT, Non-transfusion dependent thalassemia; HTN, Hypertension; DM, Diabetes mellitus; HBV, Hepatitis B virus; HCV, Hepatitis C virus; PTH, parathyroid hormone; IGF-1, Insulin-like growth factor 1.

### Outcomes

**Table 2** shows the univariate logistic regression analysis of each variable and the associated outcome. The transfusion dependency indicated double the odds of low BMD when compared with non-transfusion dependency (OR 2.36; 95%CI 1.33 to 4.21; p=0.003). Other variables associated with low BMD were a history of splenectomy (OR 3.60; 95%CI 2.00 to 6.47; p<0.001) and serum phosphate levels (OR 1.84; 95%CI 1.07 to 3.17; p=0.026). Protective factors for low BMD were higher hemoglobin levels (OR 0.78; 95%CI 0.62 to 0.99; p=0.041), higher serum albumin (OR 0.47; 95%CI 0.24 to 0.91; p=0.026) and higher BMI (OR 0.71; 95%CI 0.61 to 0.82; p <0.001).

**Table 2:** Univariate and multivariate logistic regression.

	Univariate analysis			Multivariate analysis		
	OR	95% CI	p value	OR	95% CI	p value
**Age (year)**	0.93	0.90–0.96	<0.001			
**Gender, Male**	1.57	0.87–2.81	0.129			
**BMI (kg/m2), n= 207**	0.71	0.61–0.82	<0.001	0.71	0.61–0.82	<0.001
Type of thalassemia						
• **TDT**	2.36	1.33–4.21	0.003	2.36	1.28–4.38	0.006
• **Beta thalassemia**	2.12	1.04–4.32	0.037			
**Splenectomy**	3.60	2.00–6.47	<0.001			
**Smoking**	3.83	0.19–75.60	0.377			
**Alcohol drinker**	2.20	0.24–20.21	0.485			
Comorbidity						
• **Abnormal hormone (Hypogonadism, Hypothyroid, Adrenal insufficiency)**	0.32	0.09–1.17	0.087			
• **Non-communicating disease (HTN, DM)**	1.71	0.52–5.59	0.368			
• **Chronic viral hepatitis infection (HBV, HCV)**	1.19	0.46–3.10	0.708			
• **Pulmonary hypertension**	2.24	0.71–7.07	0.168			
**Iron chelation**	2.12	0.99–4.53	0.052			
**Steroid**	2.98	0.97–9.17	0.056			
Blood examination						
• **Hemoglobin (g/dl)**	0.78	0.62–0.99	0.041			
• **Hematocrit (%)**	0.93	0.88–1.00	0.056			
• **Creatinine (mg/dl), n= 206**	0.60	0.20–1.78	0.366			
• **Ferritin (mg/L)**	1.08	0.90–1.28	0.382			
• **Serum albumin (g/dl), n= 209**	0.47	0.24–0.91	0.026			
• **Calcium (mg/dl), n= 167**	0.69	0.32–1.47	0.348			
• **Phosphate (mg/dl), n= 165**	1.84	1.07–3.17	0.026			
• **Vitamin D level (ng/ml), n= 107**	0.99	0.95–1.03	0.918			
• **PTH (pg/ml), n= 79**	1.00	0.99–1.00	0.903			
• **IGF1 (ng/ml), n= 80**	0.99	0.97–1.01	0.719			
**History of extramedullary hematopoiesis**	1.45	0.53–3.95	0.462			

BMI, Body mass index; TDT, Transfusion dependent thalassemia; NTDT, Non-transfusion dependent thalassemia; HTN, Hypertension; DM, Diabetes mellitus; HBV, Hepatitis B virus; HCV, Hepatitis C virus; PTH, parathyroid hormone; IGF-1, Insulin-like growth factor 1.

In the multivariable logistic regression model, BMI and transfusion-dependency were found to be the variables independently associated with low BMD, OR 0.71; 95%CI 0.61 to 0.82; p<0.001 and OR 2.36; 95%CI 1.28 to 4.38; p=0.006, respectively, as is shown in **Table 2**.

In the low BMD subgroup, multivariate analysis showed that variables associated with a lower Z-score were hypogonadism, adrenal insufficiency, serum phosphate and low serum IGF-1, as is shown in **Supplementary Table 1**. However, this study found no significant relationship between vitamin D levels and Z-score. In the multiple linear regression model, we analyzed the associated factor separately. It was found that in the case of the femoral neck, a low serum IGF-1 level was associated with a lower Z-score (β = -0.42; 95%CI -0.83 to -0.01; p=0.040), while an increase in serum phosphate was associated with an increase in the Z-score (β=0.40; 95%CI 0.07 to 0.73; p=0.016). In addition, only hypogonadism was related to a lower Z-score at the lumbar spine (β=-0.48, 95%CI -0.91 to -0.04, p=0.031).

## Discussion

Bone complications, including osteoporosis, are one of the most common complications in both TDT and NTDT patients. Our study confirmed a prevalence of 62.4% for these complications. We also identified the predisposing factors for low BMD in these patients, which combined the clinical type of thalassemia and the relevant laboratory data. These risk factors will help in identifying high-risk patients who require further investigation and treatment.

This study aimed to identify the prevalence of low BMD in thalassemic patients as the primary endpoint. The secondary endpoint was to identify factors predisposing low BMD values. The study found a relationship between transfusion-dependent status, some hormonal laboratory values and the Z-score. The subgroup analysis of low BMD indicated that each variable was associated with a lower Z-score at different sites. Hypogonadism was related to a lower Z-score in the lumbar spine, while low serum IGF-1 levels and changes in serum phosphate levels only affected the femoral neck. These results were consistent with those of previous studies (5, 15–18).

The prevalence of low BMD in our study was 62.4%, which is similar to the prevalence of thalassemia-associated osteoporosis (TAO) reported by Hashemieh, et al. (65.6%) (21). Jensen CE, et al. reported a higher prevalence of low BMD at 96.3%, which was then categorized into very low BMD at 51.0% and low BMD at 45.0%. However, the Jensen study included TDT beta-thalassemia major patients, which were determined to be clinically more severe (10). The differences in prevalence could be explained by the different populations that had been studied. Our study consisted of both TDT and NTDT patients, for which NTDT patients comprised about one-third of the population. Importantly, the genotype of thalassemia varies worldwide. In Thailand, there is a high prevalence of alpha-thalassemia and beta-thalassemia/HbE patients, both of whom have been found to be less clinically severe than thalassemia major patients (22). Another point to consider is the definition and cut-off point of the Z-scores in each study, which also differed. Previous studies defined osteopenia as a Z-score between -1.0 and -2.5, while osteoporosis was identified by a Z-score below -2.5 according to the WHO 1994 criteria (10, 19).

In this study, the univariable analysis indicated that factors associated with lower BMD values were older age, low BMI, low hemoglobin levels, splenectomy and transfusion dependency status, which were consistent with the factors that had been identified in previous reports (20, 23, 24). Marina Baldini et al. reported that BMD deficit in beta thalassemia major subjects occurred in up to 92.7% of patients, despite these patients having received adequate transfusions. Moreover, osteoporosis was mainly presented on the lumbar spine, while osteopenia mostly affected the femur (25). Teawtrakul N et al. also reported that the risk factors associated with vertebral fractures were low BMD, low trabecular bone scores and a history of splenectomy (5). The study conducted by Yu-Guang Chen et al. reported a higher incidence of fractures in thalassemia patients than in non-thalassemia patients with a hazard ratio of 1.35. That study also found that thalassemic patients without osteoporosis, when compared with a normal population, had a higher risk of fracture with a hazard ratio of 1.42 (26). All of these factors were likely correlated with the severity of the disease, which was confirmed by multivariable analysis in our study showing that transfusion-dependent thalassemia was the most significant factor. Lower BMI is associated with poor nutritional status, which could be a risk factor for low BMD values. In our study, there were no associations with gender, smoking, consumption of alcohol and serum ferritin levels, which is consistent with the outcomes of previous reports (10, 27, 28). The etiology might differ between studies because there are multifactorial risks between osteoporosis and low BMD values.

In the subgroup analysis, our study indicated the importance of serum markers in predicting low BMD values. Low serum IGF-1 levels were found to be a significant risk factor for a low Z-score. Ebeling PR et al. reported that bone growth and mineralization were effects of osteoblasts stimulated by IGF-1 (29). Furthermore, a study conducted by Mahachoklertwattana P et al. determined that lower serum IGF-1 levels were more likely to be found in transfusion-dependent patients in comparison to non-transfusion-dependent patients, which would then lead to lower BMD values (30). Therefore, low serum IGF-1 levels could be considered a cause of low BMD. In this group further MRI of pituitay should be done to confirm growth hormone deficiency.

Phosphate homeostasis is essential for bone mineralization, mainly through fibroblast growth factor 23 (FGF23), parathyroid hormone and vitamin D. High serum phosphate stimulates FGF23 production by osteocytes, which increases phosphate renal excretion and reduces active vitamin D production. This results in lower phosphate and calcium absorption in the intestine, which then leads to a reduction in bone mineralization (31). An increase in serum phosphate and a decrease in serum calcium also result in an increase in parathyroid hormone secretions, which increases renal calcium reabsorption, inhibits phosphate reabsortion and also results in bone resorption. Excess serum phosphate also binds to calcium, resulting in a decrease in calcium for bone mineralization, leading to a lower degree of BMD (32). The cross-sectional study conducted by Lee AW et al. found that high phosphate intake was associated with increased BMD values with adequate calcium intake in a normal population (33). Takeda E et al.’s study showed no effect of short-term high phosphate intake on FGF23; however, long-term high phosphate intake and long-term hyperphosphatemia were associated with increased incidences of osteoporosis (34). A later study conducted by Michigami T et al. reported a positive correlation between increased serum phosphate levels and an increase in FGF23, which consequently increased phosphate excretion via the kidney resulting in demineralization of the bones (31). Campos-Obando N et al. also found a correlation between increased serum phosphate levels and fracture risk (35). However, our study found an association between increased serum phosphate and an increase in the Z-score at the femoral neck. Notably, the association between phosphate and bone metabolism will require further study.

Regarding endocrinology, our study found evidence of hypogonadism and a worsening Z-score at the lumbar spine from simple linear regression analysis, which is consistent with the outcomes of previous reports (9, 36). Fung EB et al. reported a higher fracture-prevalence in hypogonadal patients with a higher prevalence in males than females (37). Another study conducted by Anapliotou ML et al. found that hypogonadism was related to lower spinal BMD; however, in this case bone density could be increased by hormonal replacement therapy (38). Accordingly, a study conducted by Baldini M et al. found that hypogonadal patients had lower femoral BMD than eugonadal patients despite adequate hormonal replacement therapy, whereas there were no significant differences in spinal BMD (25). Scacchi M. et al. found a significantly lower T-score on the femoral bone in hypogonadal thalassemic patients when compared with those characterized as eugonadal, indicating predominant demineralization in the femur bone in patients with hypogonadism (39). Based on the studies mentioned above, low BMD can be explained by bone demineralization caused by hypogonadism.

The strengths of this study include a focus on the variations in thalassemia type, including data obtained from both TDT and NTDT patients, and that BMD could be measured using the standard DXA, which is available in most hospitals as a standard. The limitations of this study include its retrospective design, which resulted in limited data availability for important factors and novel bone markers such ALP, periostin, cathepsin K (catK), receptor activator of nuclear factor kappa beta ligand (RANK-L), Dickkopf-1 (Dkk1), sphingosine-1-phosphate (S1P), osteocalcin, sclerostin, fibroblast growth factor 23 (FGF23), klotho and miRNAs. In addition, the generalizability of the study was limited due to its study design. The endocrinopathy complications and secondary osteoporosis need to be systemmatic evaluate and standard treatment since these complications may contribute to the prevalence of low BMD especially growth hormone deficiency. Further study should be further investigated on the novel markers and bone architecture to find out the pathophysiology of low BMD in this population. This may emphasize future treatment to prevent this complication.

## Conclusion

The prevalence of low BMD in this study was 62.4%. Factors associated with low BMD in thalassemic patients were transfusion-dependent status and BMI. In the low BMD subgroup, hypogonadism was associated with a lower Z-score at the lumbar spine, while serum phosphate and low serum IGF-1 levels were associated with a lower Z-score at the femoral neck.

## Data availability statement

The data that support the findings of this study are not publicly available due to privacy or ethical restrictions. This data are available from the corresponding author, AT, adisak.tan@cmu.ac.th upon reasonable request.

## Ethics statement

The studies involving humans were approved by the Institutional Research Ethics Committee at the Faculty of Medicine, Chiang Mai University (Study number: MED-2564-08128). The studies were conducted in accordance with the local legislation and institutional requirements. Written informed consent for participation was not required from the participants or the participants’ legal guardians/next of kin in accordance with the national legislation and institutional requirements.

## Author contributions

NA: Conceptualization, Data curation, Formal analysis, Investigation, Methodology, Writing – original draft. AT: Conceptualization, Formal analysis, Funding acquisition, Investigation, Methodology, Supervision, Writing – original draft, Writing – review & editing. TP: Writing – review & editing. NH: Writing – review & editing. PP: Writing – review & editing. TR: Writing – review & editing. SH: Writing – review & editing. CC: Writing – review & editing. ER: Writing – review & editing. LN: Writing – review & editing. KF: Writing – review & editing. PC: Writing – review & editing.
