# Supplementary material for: Prevalence and risk factors predisposing low bone mineral density in patients with thalassemia
Source: Front Endocrinol (Lausanne). 2024 Jun 24;15:1393865. doi: 10.3389/fendo.2024.1393865 (PMC11228236; doi:10.3389/fendo.2024.1393865)
Supplement: Supplementary file 1 [file Table_1.docx]

**Supplementary Appendix**

**Table S1** Subgroup hormone analysis of each variable to BMD Z-score at L-spine and femoral neck

|  | BMD Z-score (L spine) | | | | | | BMD Z-score (femoral neck) | | | | | | |
| --- | --- | --- | --- | --- | --- | --- | --- | --- | --- | --- | --- | --- | --- |
|  | Simple linear regression | | | Multiple linear regression | | | Simple linear regression | | | Multiple linear regression | | | |
|  | β | 95% CI | p value | β | 95% CI | P value | β | 95% CI | p value | β | 95% CI | p value |  |
| Endocrine complication, n (%)   - Diabetes mellitus, n= 210 - Hypogonadism, n=98 - Hypothyroidism, n= 199 - Adrenal insufficiency, n= 210 | -0.40  -0.41  -0.01  -0.65 | -1.04, 0.25  -0.76, -0.05  -0.38, 0.36  -1.26, -0.04 | 0.228  0.024  0.954  0.036 | -0.48  0.18 | -0.91, -0.04  -0.42, 0.78 | 0.031  0.553 | -0.39  -0.40  -0.14  -0.60 | -0.94, 0.15  -0.77, -0.03  -0.46, 0.17  -1.11, -0.09 | 0.157  0.031  0.364  0.021 | -0.29  -0.53 | -0.75, 0.15  -1.16, 0.10 | 0.197  0.099 |  |
| Blood examination   - Calcium (mg/dl), mean±sd - Phosphate (mg/dl), mean±sd - Serum albumin (g/dl) - Insufficiency to deficiency   serum 25(OH)D (ng/ml), n= 107   - High PTH level (pg/ml), n= 79 - Low IGF1 (ng/ml), n= 80 | 0.28  -0.30  0.63  0.14  -2.12  -0.33 | -0.07, 0.65  -0.52, -0.07  0.29, 0.98  -0.28, 0.56  -3.55, -0.69  -0.71, 0.03 | 0.122  0.010  <0.001  0.514  0.004  0.079 | -0.02  -0.27 | -0.33, 0.28  -0.66, 0.10 | 0.861  0.150 | 0.29  -0.07  0.67  0.09  -3.50  -0.36 | -0.00, 0.59  -0.26, 0.12  0.38, 0.95  -0.30, 0.49  -5.09, -1.92  -0.77, 0.04 | 0.055  0.462  <0.001  0.639  <0.001  0.083 | 0.40  -0.42 | 0.07, 0.73  -0.83, -0.01 | 0.016  0.040 |  |
